# Public perception of medical detection dogs and other COVID-19 testing strategies

**DOI:** 10.3389/fpubh.2025.1641243

**Published:** 2025-09-04

**Authors:** Karolina Zacharias, Sebastian Meller, Nele A. ten Hagen, Holger A. Volk, Friederike Twele

**Affiliations:** ^1^Department of Small Animal Medicine and Surgery, University of Veterinary Medicine Hannover, Hannover, Germany; ^2^Center for Systems Neuroscience Hannover, Hannover, Germany

**Keywords:** COVID-19, SARS-CoV-2 detection, medical detection dogs, opinion survey, antigen rapid tests, polymerase chain reaction, pandemic preparedness

## Abstract

The COVID-19 pandemic led to social restrictions, severely impacting many sectors, including the cultural and gastronomic sectors. To restore normality, various testing approaches were proposed, but public opinion and acceptance of these tests in Germany had not been investigated. Proposed solutions included antigen rapid diagnostic tests (Ag-RDTs), polymerase chain reaction (PCR) tests, and medical detection dogs. The aim of the current study was to assess public perceptions regarding the use of canines for SARS-CoV-2 detection. A feasibility study on SARS-CoV-2 detection using trained detection dogs was conducted among 2,802 concertgoers across four events. Participants aged 18 years and older, provided informed consent, shared their medical history, and completed a survey on various testing methods. They then underwent PCR testing, Ag-RDTs, and canine testing. After the concerts, 1,315 participants completed a follow-up survey about their experiences with the different testing systems. Before the concerts, 70% of respondents preferred using dogs for testing, with 32% favoring direct sniffing and 39% preferring sweat sample testing. After the concert, 72% still preferred canine testing, with 40% voting for direct sniffing and 32% for indirect sweat sample testing. Twenty-one percent preferred PCR testing before the concerts and 23% afterwards. Respondents also recommended deploying medical detection dogs at large events, such as concerts, and at transportation hubs. However, opinions were divided on their use for SARS-CoV-2 screening in schools. These results underscore the importance of context-specific testing strategies and standardized guidelines for canine detection. While many participants preferred direct sniffing, the sweat sample method emerged as a more practical and privacy-conscious alternative. The study provides valuable insights into public acceptance of various testing methods and emphasizes the potential of canine testing at large events. Clear guidelines and proper training of detection dogs will be crucial for future pandemic preparedness.

## Introduction

The COVID-19 pandemic led to significant changes worldwide. At its onset, the absence of a vaccine and accessible testing methodologies left populations vulnerable to SARS-CoV-2 infection. In response, strict social restrictions were imposed, including lockdowns beginning in Germany on March 22, 2020, which prohibited public gatherings such as concerts, sports events, and family meetings (1, 2). These measures were accompanied by the rapid development and deployment of various testing methodologies aimed at controlling viral transmission.

The resulting void in social interactions created challenges for many sectors, particularly the cultural and hospitality industries (3, 4). To facilitate the safe reintegration of social activities, several strategies were introduced, including the use of Ag-RDTs, PCR tests, medical detection dogs, and the deployment of new vaccines (5–11). Despite high acceptance of testing technologies, studies revealed that uncertainty about eligibility, logistical issues such as accessing test sites, discomfort during sample extraction, and concerns about the consequences of a positive result were significant barriers to testing uptake (12, 13). In workplaces, temperature screening was introduced as a preventive measure, requiring employees to undergo checks before entering the premises (14, 15).

The PCR test, widely recognized as the gold standard for its exceptional sensitivity and specificity (7, 16–19), faces practical limitations in everyday use due to its reliance on sophisticated equipment, high costs, and the typical processing times of 1–2 days (17, 20). While a more time-efficient variant is available, it still necessitates 2–5 h to complete (21). The PCR test typically relies on nasopharyngeal or oropharyngeal swab samples, which require trained personnel for accurate and safe sample collection (22). Nasopharyngeal swabs involve the insertion of a long, flexible stick deep into the nasal cavity to collect viral material from the upper part of the throat caudal to the nose, while oropharyngeal swabs target the back of the throat via the mouth. Although effective, both techniques can cause discomfort, such as gagging, sneezing, or slight bleeding, especially in repeated testing contexts (23, 24). Pain and discomfort during testing procedures are well-documented barriers that contribute to lack of willingness and reduced participation in mass testing and research programs (25, 26). In Germany, as part of the citizen testing initiative, people had free access to testing centers that used Ag-RDTs (27, 28). As a result, the less reliable Ag-RDTs became more widely used (28), with PCR tests primarily employed to confirm or rule out SARS-CoV-2-positive rapid test results (27, 28). Rapid tests, while accessible, often created confusion around result interpretation and subsequent steps, such as self-isolation or returning to work, further emphasizing the need for clear communication and guidelines (28).

Further studies have reinforced the need for scalable, integrated diagnostic systems that combine high sensitivity with broad accessibility. This encompasses smartphone-compatible point-of-care technologies as well as innovative approaches such as AI-driven or sensor-based diagnostics (29).

To overcome some of the aforementioned limitations of these testing systems, many groups worldwide explored new testing methods such as canine medical detection (9–11, 30–36). Attracting interest from the World Health Organization (37), studies have shown that canine detection exhibits high sensitivity (approximately 85%) and specificity (up to 99%) in distinguishing acute SARS-CoV-2 infections from negatives (30, 33). Dogs were also effective in distinguishing samples from SARS-CoV-2-infected individuals from those infected with other respiratory pathogens (35), making them a versatile tool in pandemic management. In a feasibility study evaluating large-scale testing at concerts, dogs demonstrated a specificity of over 99% and a sensitivity of 82% (36). These results suggest that medical detection dogs are reliable in real-world scenarios, offering performance comparable to PCR tests and significantly surpassing Ag-RDTs (9, 33, 38, 39).

Moreover, the application of scent detection dogs was considerably more economical, demonstrating a substantial cost reduction compared to both PCR and Ag-RDTs (40).

COVID-19 vaccines became central to pandemic control; however, their impact depends not only on efficacy but also on rapid and widespread distribution. Barriers such as infrastructure limitations, cold chain logistics, vaccine hesitancy, and unequal access continue to constrain their effectiveness (41). Additionally host factors including age, sex, and comorbidities influence individual vaccine responses, complicating the implementation of uniform vaccination strategies (42). Heterologous vaccination regimens, such as mRNA boosters following vector-based vaccines, have demonstrated stronger immune responses and offer a practical solution during shortages (43).

This article presents the outcomes of a comprehensive public opinion survey conducted within the framework of the aforementioned feasibility study (36), aimed at gauging attendees’ perceptions and preferences regarding various SARS-CoV-2 testing methodologies used during the study. With the eventual conclusion of the COVID-19 pandemic, it is crucial to acknowledge the potential of medical detection dogs for future pandemics in our interconnected world (37). Understanding public attitudes and concerns regarding testing modalities is essential for crafting effective public health policies, optimizing events’ safety measures, and fostering public trust in these strategies.

## Methods

### Study design and ethics

**Figure 1:**
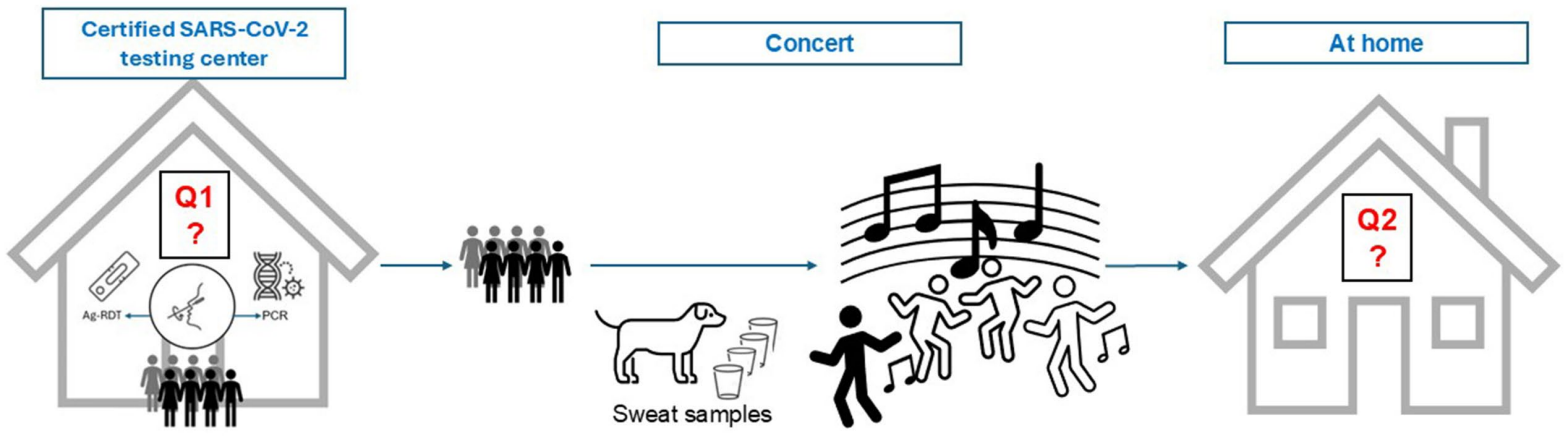
Schematic overview of the study design in chronological order from left to right. PCR, Ag-RDT, and Questionnaire 1 (Q1) were completed at a certified testing center. Upon arrival at the concert venue, participants provided a sweat sample, which was analysed for SARS-CoV-2 by trained detection dogs. Admission to the concert area was granted following a negative result. After the event, Questionnaire 2 (Q2) was completed at home via email.

**Figure 2:**
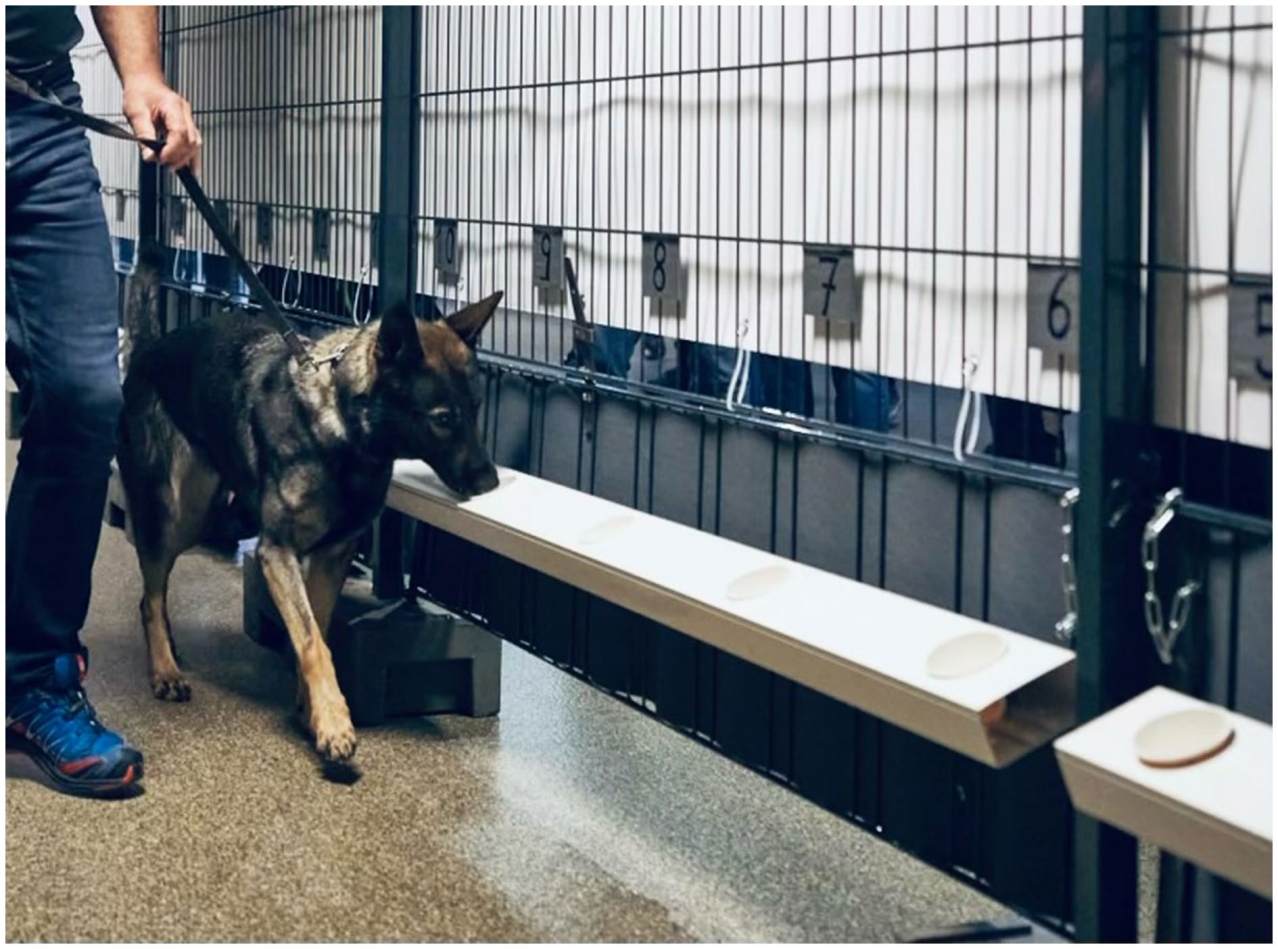
A trained detection dog at work at the concert.

As part of a feasibility study on SARS-CoV-2 detection using trained detection dogs (36), concert attendees were surveyed before and after the events. The study followed a three-phase design (Figure 1): first, participants visited a certified SARS-CoV-2 testing center, where they underwent both PCR and antigen testing and completed an initial questionnaire. Second, eligible individuals attended one of four concerts and, prior to admission, provided a sweat sample from the crook of the arm (antecubital fossa), which was screened for SARS-CoV-2 by trained detection dogs (Figure 2). Third, after the event, participants were invited to complete a follow-up questionnaire at home, reflecting on their experience and perceptions of the testing methods.

The study was conducted in compliance with the ethical standards outlined by the Declaration of Helsinki and received approval from the local Ethics Committee of Hannover Medical School (MHH) (ethics consent numbers 9042_BO_K_2020 and 9940_BO_S_2021). Both the study and the concerts were officially authorized by local health and regulatory authorities. Written informed consent was obtained from all participants prior to sample collection, and animal testing was approved by the German Armed Forces.

### Pre-concert testing and initial data collection

Initially, all participants visited a certified SARS-CoV-2 testing center. There, they provided written informed consent, underwent antigen rapid diagnostic testing (Ag-RDTs) and PCR testing, and completed a preliminary questionnaire.

### Questionnaire 1: pre-concert assessment

The initial questionnaire consisted of two sections: the first gathered general demographic and medical data relevant for evaluating canine detection performance, including age, gender, vaccination status, medical history, and current medications. The second section addressed participants’ perceptions of different SARS-CoV-2 testing methods, such as canine detection, PCR tests, Ag-RDTs (at official testing centers), and self-administered Ag-RDTs. Participants rated their level of confidence in each method on a five-point Likert scale ranging from 1 (full confidence/completely agree) to 5 (no confidence/completely disagree).

A total of 4,124 individuals responded to the first questionnaire, although not all who registered for the concerts ultimately attended. To qualify, attendees had to be at least 18 years old and reserve free personalized tickets. Individuals involved in the training phase of the detection dogs were excluded to avoid potential recognition bias.

### Concert attendance

A total of 2,802 concert-goers attended one of four concerts, which were held 5–8 days apart. Attendance varied across events: 466 participants attended the first concert, 640 at the second, 678 at the third, and 1,018 at the final event. Prior to entry, all participants provided a sweat sample from the crook of the arm, which was screened for SARS-CoV-2 by trained medical detection dogs.

### Questionnaire 2: post-concert assessment

Following the concerts, a second questionnaire was distributed via email to participants, allowing them to reflect on their experience with the canine detection process. This survey included basic demographic items to support analysis and focused on participants’ views regarding the broader use of detection dogs for COVID-19 detection.

Respondents were asked where they would consider the deployment of detection dogs appropriate, with options including airports, schools, healthcare settings, sporting events, and workplaces. Additional questions assessed participants’ willingness to be tested by a dog, preferences regarding direct contact versus indirect testing, and their confidence in each testing method.

A total of 1,315 individuals completed the second questionnaire. In accordance with ethical and General Data Protection Regulation standards, responses to this follow-up survey were anonymous.

### Data analysis

Descriptive statistics were used to analyze the data, and only fully completed responses were included to ensure accuracy. Data were organized using Microsoft® Excel® for Microsoft 365 MSO (Version 2,504, Build 16.0.18730.20186, 64-bit), part of the Microsoft 365 Apps for Enterprise suite (Microsoft Corporation, Redmond, WA, USA), facilitating efficient visualization and analysis of key trends.

Confidence intervals for proportions were calculated using the binomial method via an online tool from Sample-Size.net (https://sample-size.net/confidence-interval-proportion/), based on JavaScript functions developed by John C. Pezzullo.

## Results

### Characterization of participants

The study included a diverse group of respondents across gender and age categories (see Table 1). A total of 5,439 questionnaires were completed, with 4,124 before the events and 1,315 afterwards. The majority of participants were between 21 and 40 years of age, followed by those aged 41–65. Responses were received from male and female participants, and a small number of participants identifying as diverse, with some individuals omitting gender or age information. Table 1 provides a detailed breakdown of responses by gender and age across both time points.

**Table 1:** Characterization of participating individuals.

Age (in years)	Gender	Pre concert		Post concert	
		Counts	Percentage	Counts	Percentage
<20		116	2.81%	57	4.33%
	Diverse	1	0.02%	1	0.08%
	Male	31	0.75%	20	1.52%
	Female	84	2.04%	38	2.89%
	No answer	0	0.00%	18	1.37%
21–40		2,423	58.75%	725	55.13%
	Diverse	2	0.05%	0	0.00%
	Male	895	21.70%	236	17.95%
	Female	1,526	37.00%	489	37.19%
41–65		1,538	37.29%	513	39.01%
	Diverse	2	0.05%		0.00%
	Male	594	14.40%	183	13.92%
	Female	942	22.84%	330	25.10%
>65		47	1.14%	10	0.76%
	Male	28	0.68%	8	0.61%
	Female	19	0.46%	2	0.15%
Total		4,124	100%	1,315	100%

The demographic distribution in Table 1 shows that the majority of participants were between 21 and 40 years old, accounting for 58.75% before the concert and 55.13% afterwards. This age group was followed by individuals aged 41–65. Across all age categories, female respondents made up the largest proportion.

### Preferences for different COVID-19 testing methods

**Figure 3:**
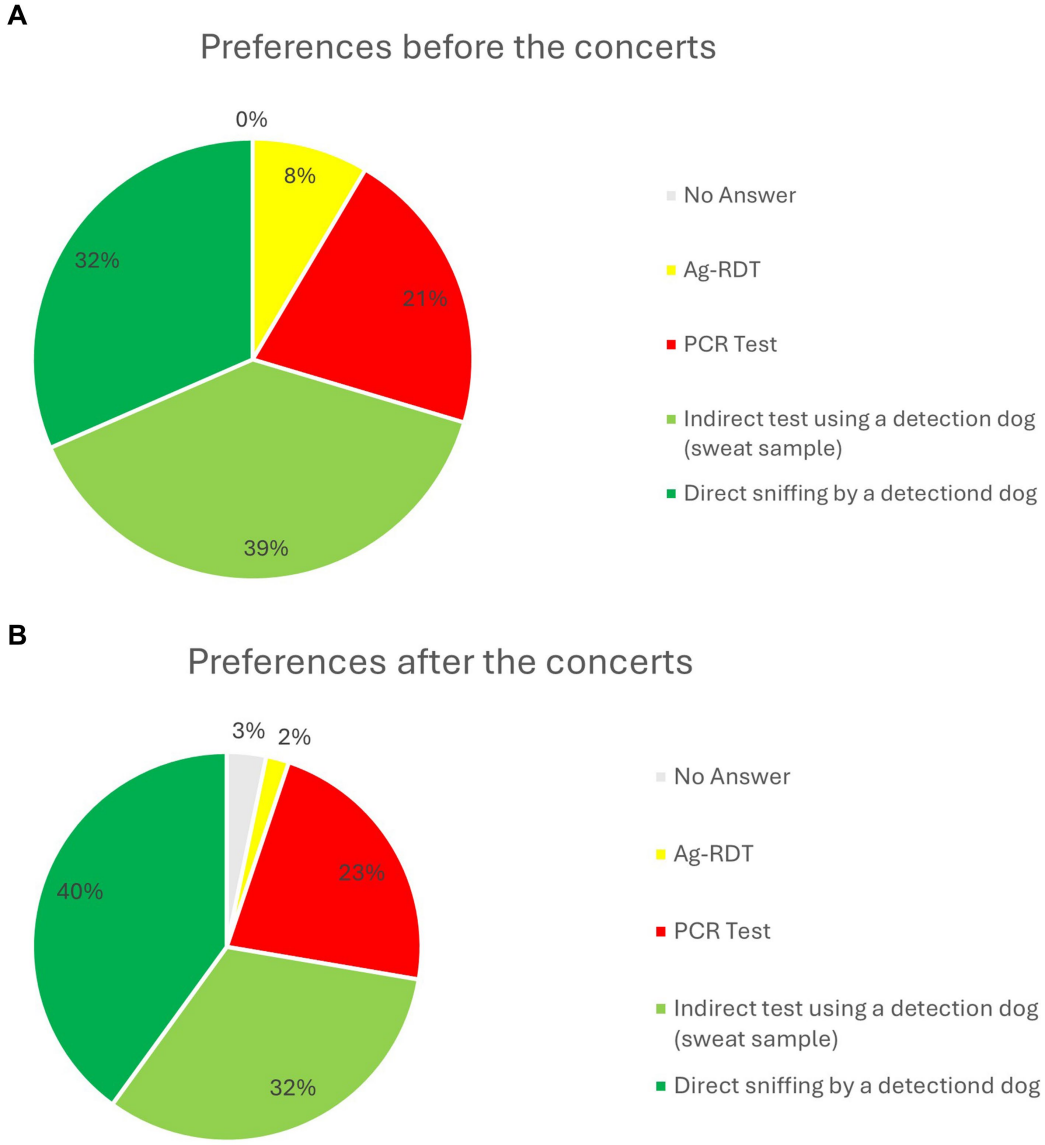
**(A)** Preferred testing methods before the concert in percentage: No answer (gray), Ag-RDTs (yellow), PCR test (red), indirect test using a detection dog (light green), and direct sniffing by a detection dog (green). **(B)** Preferred test methods after he concert in percentage: No Answer (gray), Ag-RDTs (yellow), PCR test (red), indirect test using a detection dog (light green), and direct sniffing by a detection dog (green).

Before a concert, 8.54% preferred Ag-RDTs, 21.07% PCR test, 31.55% direct sniffing by a detection dog, and 38.85% the indirect sweat sample test using a detection dog. After the concert, these preferences shifted, with only 1.9% opting for Ag-RDTs, 22.51% for PCR test, 40% for direct sniffing by the dogs, and 32.32% for indirect sweat sample test using a detection dog. 3.27% refrained from expressing an opinion. These results (Table 2) are visualized in Figures 3A,B and demonstrate a marked increase in the preference for direct sniffing after concert participation (+8.45%), while the preference for sweat samples slightly declined (−6.53%) and a sharp drop in was observed preference for the Ag-RDTs (−6,64%).

**Table 2:** Preferences for different COVID-19 testing methods before and after the concerts.

Strategy	Pre concert				Post concert			
	Counts	Percentage	Lower CI	Upper CI	Counts	Percentage	Lower CI	Upper CI
Ag-RDT	352	8.54%	7.70%	9.43%	25	1.90%	1.23%	2.79%
PCR test	869	21.07%	19.84%	22.35%	296	22.51%	20.28%	24.87%
Direct sniffing by a detection dog	1,301	31.55%	30.13%	32.99%	526	40.00%	37.34%	42.71%
Sweat sample test using a detection dog	1,602	38.85%	37.35%	40.35%	425	32.32%	29.79%	34.92%
No answer	0	0%	0%	0%	43	3.27%	2.38%	4.38%
Total	4,124	100.00%			1,315	100.00%		

### Confidence in PCR testing before and after the concert

The questionnaire prior to the concert revealed that PCR tests were highly acknowledged, with 47.62% of participants having a high level of trust in them, and an additional 39.38% considering them reliable (Table 3). In contrast, 1.24% of participants had little confidence, and only 0.32% had no confidence in PCR tests, while 11.45% were neutral.

**Table 3:** Attendees confidence in polymerase chain reaction test before and after the concerts.

	Pre concert				Post concert			
	Counts	Percentage	Lower CI	Upper CI	Counts	Percentage	Lower CI	Upper CI
No answer	0	0.00%	0.00%	0.00%	40	3.04%	2.18%	4.12%
No confidence	13	0.32%	0.17%	0.54%	3	0.23%	0.05%	0.67%
Little confidence	51	1.24%	0.92%	1.62%	11	0.84%	0.42%	1.49%
Neutral	472	11.45%	10.49%	12.46%	119	9.05%	7.55%	10.73%
Confidence	1,624	39.38%	37.88%	40.89%	520	39.54%	36.89%	42.25%
High confidence	1964	47.62%	46.09%	49.16%	622	47.30%	44.57%	50.04%
Total	4,124	100%			1,315	100%		

**Figure 4:**
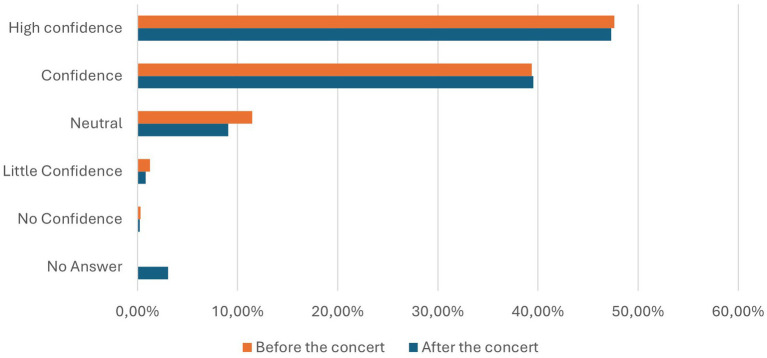
Confidence of the participants considering the PCR testing method before (orange) and after (blue) the concert in percentage.

Following the concert, only 0.23% of respondents reported having no confidence in PCR tests, while 0.84% expressed little confidence. A neutral stance was held by 9.05%, whereas 39.54% regarded PCR tests as reliable and 47.30% considered them highly reliable. An additional 3.04% chose not to provide an opinion. These results are detailed in Table 3, which presents a breakdown of confidence levels in PCR testing before and after the concert, and are illustrated in Figure 4, providing a visual representation of participants’ responses during both phases of the study.

### Shifts in perception of dogs as a COVID-19 testing method pre- and post-concert

**Figure 5:**
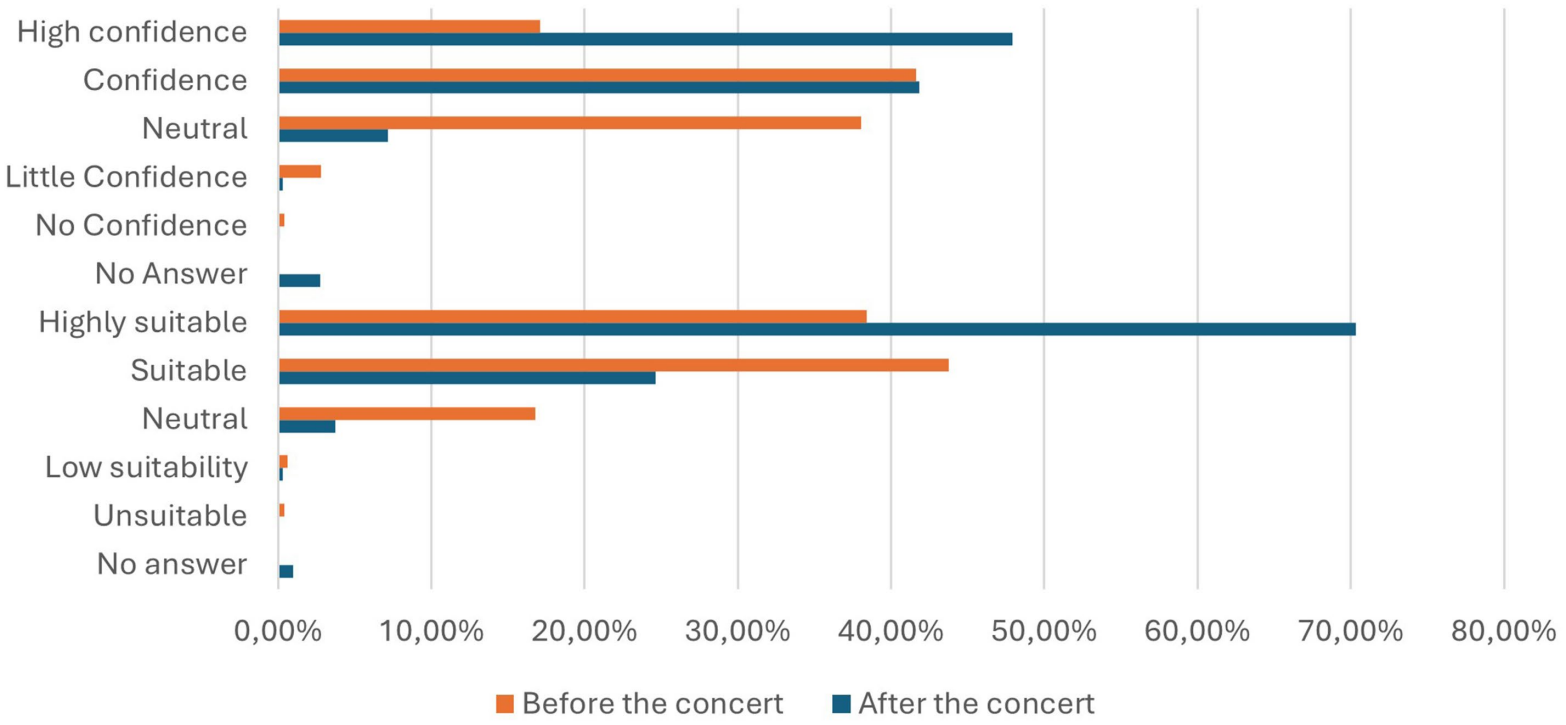
Opinion of the participants regarding their confidence level (above) and the suitability (below) regarding detection dogs as a testing method before (orange) and after (blue) the concert in percentage.

**Figure 6:**
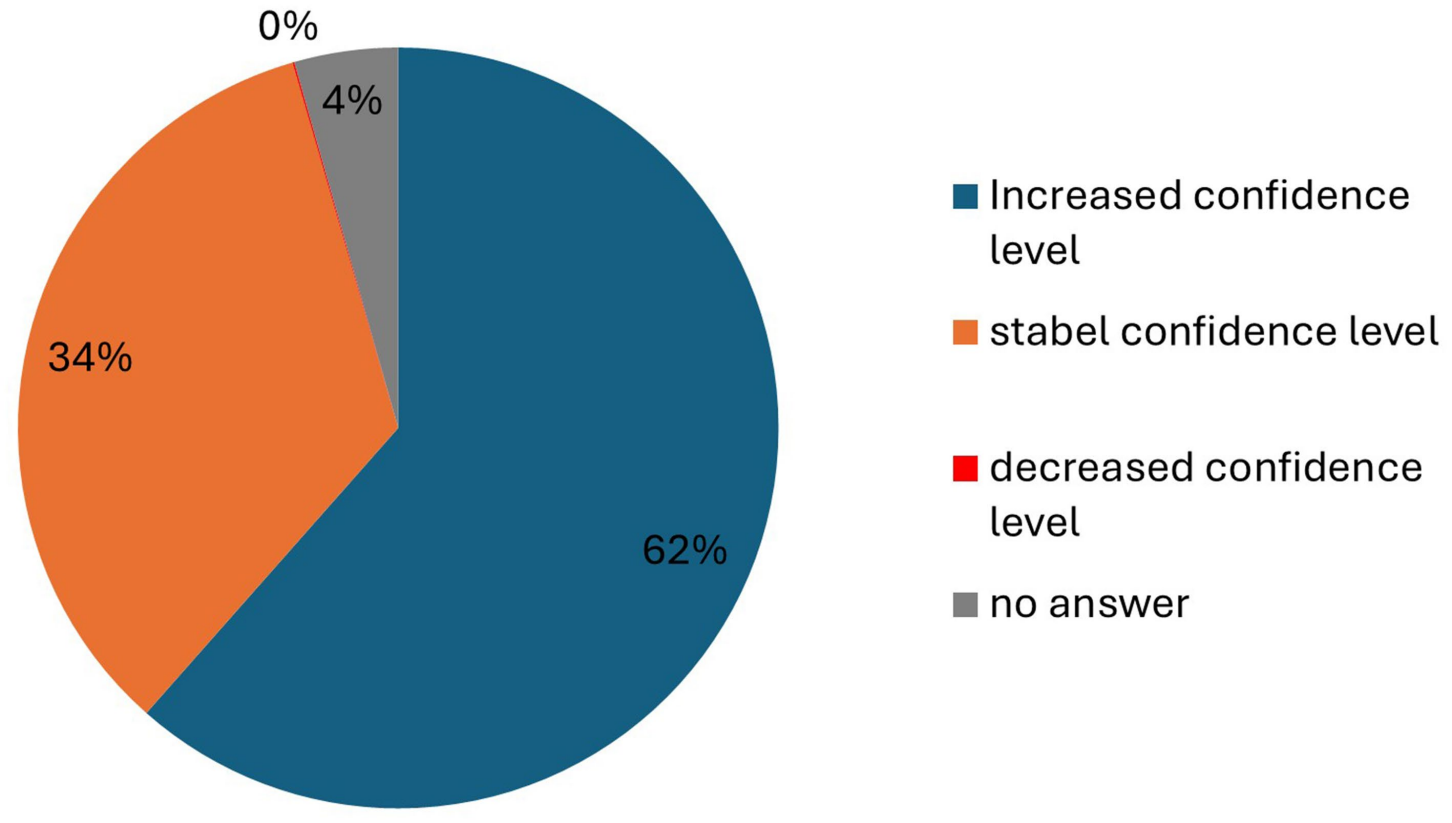
Stability of participants’ confidence level regarding detection dogs as a SARS-CoV-2 testing method. Increased confidence level (blue), stable confidence level (orange), decreased confidence level (red), and no answer (gray).

**Figure 7:**
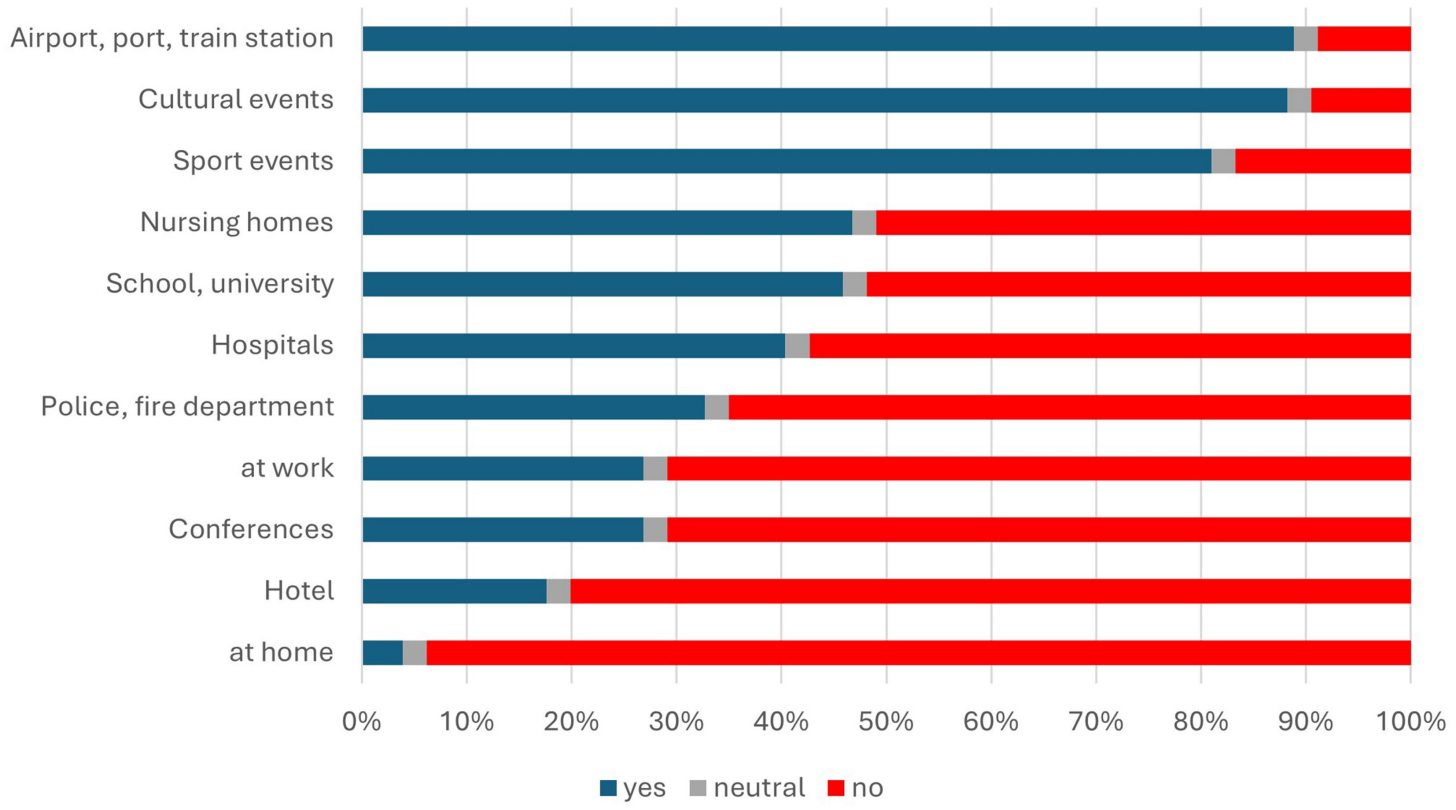
Respondents’ opinions on potential deployment locations for sniffer dogs with three response options: yes (blue), no (red), and neutral (gray).

Participants were presented with multiple questions regarding detection dogs. They were asked both before and after the concert whether they considered detection dogs suitable for identifying SARS-CoV-2 infected individuals and how much confidence they had in this method (Figure 5 and Table 4). Additionally, participants were questioned about whether their attitudes had changed after experiencing the entry procedure at the concert (Figure 6 and Table 4) and where they believed the use of detection dogs would be appropriate (Figure 7).

**Table 4:** Opinions on suitability, confidence level and changes in confidence level of attendees concerning detection dogs.

	Pre concert				Post concert			
	Counts	Percentage	Lower CI	Upper CI	Counts	Percentage	Lower CI	Upper CI
No answer	0	0.00%	0.00%	0.00%	13	0.99%	0.53%	1.68%
Unsuitable	17	0.41%	0.24%	0.66%	0	0.00%	0.00%	0.00%
Low suitability	25	0.61%	0.39%	0.89%	4	0.30%	0.08%	0.78%
Neutral	693	16.80%	15.67%	17.98%	49	3.73%	2.77%	4.90%
Suitable	1805	43.77%	42.25%	45.30%	324	24.64%	22.33%	27.06%
Highly suitable	1,584	38.41%	36.92%	39.91%	925	70.34%	67.79%	72.80%
Total	4,124	100.00%			1,315	100.00%		
No answer	0	0.00%	0.00%	0.00%	36	2.74%	1.92%	3.77%
No confidence	17	0.41%	0.24%	0.66%	1	0.08%	0.00%	0.42%
Little confidence	115	2.79%	2.31%	3.34%	4	0.30%	0.08%	0.78%
Neutral	1,570	38.07%	36.58%	39.57%	94	7.15%	5.81%	8.68%
Confidence	1717	41.63%	40.12%	43.16%	550	41.83%	39.14%	44.55%
High confidence	705	17.10%	15.96%	18.28%	630	47.91%	45.18%	50.65%
Total	4,124	100%			1,315	100%		
No answer					58	4.41%	3.37%	5.66%
Decreased confidence level					1	0.08%	0.00%	0.42%
Stable confidence level					447	33.99%	31.43%	36.62%
Increased confidence level					809	61.52%	58.83%	64.16%
Total					1,315	100%		

#### Before the concert

Among participants, 38.41% considered detection dogs highly suitable for identifying SARS-CoV-2 infections, while 43.77% deemed them suitable. A neutral stance was held by 17.8%, with 0.61% regarding the dogs as somewhat unsuitable and 0.41% opposing their use entirely. These perceptions aligned closely with participants’ confidence in the reliability of detection dogs. Specifically, 17.10% rated the dogs as very reliable, 41.63% as reliable, and 38.07% remained neutral. In contrast, 2.79% found the dogs somewhat unreliable, and 0.41% expressed no confidence in them.

#### After the concert

Confidence rose significantly: 47.91% rated detection dogs as very reliable and 41.83% as reliable. Neutral responses decreased to 7.15%, and less than 1% were doubtful. In terms of suitability, 70.34% rated dogs as highly suitable, and 24.64% as suitable, an overall positive rating of 94.98%.

#### Changes in confidence

Following the concert, 61.52% of participants reported increased confidence in the use of trained detection dogs, while 33.99% stated their confidence remained unchanged, and only 0.08% reported a decrease (Table 4).

#### Opinions on the use of detection dogs in various settings

Table 5 and Figure 7 provide a detailed overview of public attitudes regarding the appropriate settings for the use of detection dogs. The findings reveal strong support for their deployment in large-scale, high-traffic public venues. Specifically, 88.82% of respondents endorsed their use at airports, ports, and train stations, while similarly high levels of approval were reported for cultural events (88.21%) and sporting events (80.99%). These results suggest a broad public consensus that canine testing is both appropriate and desirable in contexts where efficient screening and crowd management are critical.

**Table 5:** Opinions of the participants regarding various potential application areas for detection dogs as a detection method.

Location	Age	Yes				Neutral				No				Total			
		Counts	Percentage	Lower CI	Upper CI	Counts	Percentage	Lower CI	Upper CI	Counts	Percentage	Lower CI	Upper CI	Counts	Percentage	Lower CI	Upper CI
At home	<20 years	7	0.53%	0.21%	1.09%	1	0.08%	0.00%	0.42%	49	3.73%	2.77%	4.90%	57	4.33%	3.30%	5.58%
	21–40 years	30	2.28%	1.54%	3.24%	16	1.22%	0.70%	1.97%	679	51.63%	48.90%	54.37%	725	55.13%	52.40%	57.85%
	41–65 years	14	1.06%	0.58%	1.78%	3	0.23%	0.05%	0.67%	496	37.72%	35.09%	40.40%	513	39.01%	36.36%	41.71%
	>65 years	0	0.00%	0.00%	0.00%	0	0.00%	0.00%	0.00%	10	0.76%	0.37%	1.39%	10	0.76%	0.37%	1.39%
	Empty	0	0.00%	0.00%	0.00%	10	0.76%	0.37%	1.39%	0	0.00%	0.00%	0.00%	10	0.76%	0.37%	1.39%
	Total	51	3.88%	2.90%	5.07%	30	2.28%	1.54%	3.24%	1,234	93.84%	92.40%	95.08%	1,315	100.00%		
Hotel	<20 years	12	0.91%	0.47%	1.59%	1	0.08%	0.00%	0.42%	44	3.35%	2.44%	4.47%	57	4.33%	3.30%	5.58%
	22–40 years	132	10.04%	8.47%	11.79%	16	1.22%	0.70%	1.97%	577	43.88%	41.17%	46.61%	725	55.13%	52.40%	57.85%
	42–65 years	87	6.62%	5.33%	8.10%	3	0.23%	0.05%	0.67%	423	32.17%	29.65%	34.77%	513	39.01%	36.36%	41.71%
	>65 years	1	0.08%	0.00%	0.42%	0	0.00%	0.00%	0.00%	9	0.68%	0.31%	1.30%	10	0.76%	0.37%	1.39%
	Empty	0	0.00%	0.00%	0.00%	10	0.76%	0.37%	1.39%	0	0.00%	0.00%	0.00%	10	0.76%	0.37%	1.39%
	Total	232	17.64%	15.62%	19.81%	30	2.28%	1.54%	3.24%	1,053	80.08%	77.81%	82.20%	1,315	100.00%		
Conference	<20 years	18	1.37%	0.81%	2.15%	1	0.08%	0.00%	0.42%	38	2.89%	2.05%	3.95%	57	4.33%	3.30%	5.58%
	23 –40 years	210	15.97%	14.03%	18.06%	16	1.22%	0.70%	1.97%	499	37.95%	35.32%	40.63%	725	55.13%	52.40%	57.85%
	43–65 years	124	9.43%	7.90%	11.14%	3	0.23%	0.05%	0.67%	386	29.35%	26.90%	31.90%	513	39.01%	36.36%	41.71%
	>65 years	1	0.08%	0.00%	0.42%	0	0.00%	0.00%	0.00%	9	0.68%	0.31%	1.30%	10	0.76%	0.37%	1.39%
	Empty	0	0.00%	0.00%	0.00%	10	0.76%	0.37%	1.39%	0	0.00%	0.00%	0.00%	10	0.76%	0.37%	1.39%
	Total	353	26.84%	24.46%	29.33%	30	2.28%	1.54%	3.24%	932	70.87%	68.34%	73.32%	1,315	100.00%		
At work	<20 years	13	0.99%	0.53%	1.68%	1	0.08%	0.00%	0.42%	43	3.27%	2.0.38%	4.38%	57	4.33%	3.30%	5.58%
	24–40 years	93	7.07%	5.75%	8.59%	16	1.22%	0.70%	1.97%	616	46.84%	44.12%	49.58%	725	55.13%	52.40%	57.85%
	44–65 years	64	4.87%	3.77%	6.17%	3	0.23%	0.05%	0.67%	446	33.92%	31.36%	36.55%	513	39.01%	36.36%	41.71%
	>65 years	0	0.00%	0.00%	0.00%	0	0.00%	0.00%	0.00%	10	0.76%	0.37%	1.39%	10	0.76%	0.37%	1.39%
	Empty	0	0.00%	0.00%	0.00%	10	0.76%	0.37%	1.39%	0	0.00%	0.00%	0.00%	10	0.76%	0.37%	1.39%
	Total	170	12.93%	11.16%	14.86%	30	2.28%	1.54%	3.24%	1,115	84.79%	82.73%	86.69%	1,315	100.00%		
Police, fire department	<20 years	20	1.52%	0.93%	2.34%	1	0.08%	0.00%	0.42%	36	2.74%	1.92%	3.77%	57	4.33%	3.30%	5.58%
	25–40 years	240	18.25%	16.20%	20.45%	16	1.22%	0.70%	1.97%	469	35.67%	33.07%	38.32%	725	55.13%	52.40%	57.85%
	45–65 years	167	12.70%	10.95%	14.62%	3	0.23%	0.05%	0.67%	343	26.08%	23.73%	28.55%	513	39.01%	36.36%	41.71%
	>65 years	3	0.23%	0.05%	0.67%	0	0.00%	0.00%	0.00%	7	0.53%	0.21%	1.09%	10	0.76%	0.37%	1.39%
	Empty	0	0.00%	0.00%	0.00%	10	0.76%	0.37%	1.39%	0	0.00%	0.00%	0.00%	10	0.76%	0.37%	1.39%
	Total	430	32.70%	30.17%	35.31%	30	2.28%	1.54%	3.24%	855	65.02%	62.37%	67.60%	1,315	100.00%		
Hospitals	<20 years	24	1.83%	1.17%	2.70%	1	0.08%	0.00%	0.42%	32	2.43%	1.67%	3.42%	57	4.33%	3.30%	5.58%
	26–40 years	290	22.05%	19.84%	24.39%	16	1.22%	0.70%	1.97%	419	31.86%	29.35%	34.46%	725	55.13%	52.40%	57.85%
	46–65 years	215	16.35%	14.39%	18.46%	3	0.23%	0.05%	0.67%	295	22.43%	20.20%	24.79%	513	39.01%	36.36%	41.71%
	>65 years	2	0.15%	0.02%	0.55%	0	0.00%	0.00%	0.00%	8	0.61%	0.26%	1.19%	10	0.76%	0.37%	1.39%
	Empty	0	0.00%	0.00%	0.00%	10	0.76%	0.37%	1.39%	0	0.00%	0.00%	0.00%	10	0.76%	0.37%	1.39%
	Total	531	40.38%	37.71%	43.09%	30	2.28%	1.54%	3.24%	754	57.34%	54.61%	60.03%	1,315	100.00%		
School, university	<20 years	30	2.28%	1.54%	3.24%	1	0.08%	0.00%	0.42%	26	1.98%	1.30%	2.88%	57	4.33%	3.30%	5.58%
	27–40 years	327	24.87%	22.55%	27.30%	16	1.22%	0.70%	1.97%	382	29.05%	26.61%	31.59%	725	55.13%	52.40%	57.85%
	47–65 years	242	18.40%	16.34%	20.61%	3	0.23%	0.05%	0.67%	268	20.38%	18.23%	22.66%	513	39.01%	36.36%	41.71%
	>65 years	4	0.30%	0.08%	0.78%	0	0.00%	0.00%	0.00%	6	0.46%	0.17%	0.99%	10	0.76%	0.37%	1.39%
	Empty	0	0.00%	0.00%	0.00%	10	0.76%	0.37%	1.39%	0	0.00%	0.00%	0.00%	10	0.76%	0.37%	1.39%
	Total	603	45.86%	43.14%	48.59%	30	2.28%	1.54%	3.24%	682	51.86%	49.12%	54.60%	1,315	100.00%		
Nursing homes	<20 years	28	2.13%	1.42%	3.06%	1	0.08%	0.00%	0.42%	28	2.13%	1.42%	3.06%	57	4.33%	3.30%	5.58%
	28–40 years	341	25.93%	23.58%	28.39%	16	1.22%	0.70%	1.97%	368	27.98%	25.57%	30.50%	725	55.13%	52.40%	57.85%
	48–65 years	242	18.40%	16.34%	20.61%	3	0.23%	0.05%	0.67%	268	20.38%	18.23%	22.66%	513	39.01%	36.36%	41.71%
	>65 years	4	0.30%	0.08%	0.78%	0	0.00%	0.00%	0.00%	6	0.46%	0.17%	0.99%	10	0.76%	0.37%	1.39%
	Empty	0	0.00%	0.00%	0.00%	10	0.76%	0.37%	1.39%	0	0.00%	0.00%	0.00%	10	0.76%	0.37%	1.39%
	Total	615	46.77%	44.04%	49.51%	30	2.28%	1.54%	3.24%	670	50.95%	48.21%	53.69%	1,315	100.00%		
Sport events	<20 years	47	3.57%	2.64%	4.72%	1	0.08%	0.00%	0.42%	9	0.68%	0.31%	1.30%	57	4.33%	3.30%	5.58%
	29–40 years	577	43.88%	41.17%	46.61%	16	1.22%	0.70%	1.97%	132	10.04%	8.47%	11.79%	725	55.13%	52.40%	57.85%
	49–65 years	436	33.16%	30.61%	35.77%	3	0.23%	0.05%	0.67%	74	5.63%	4.44%	7.01%	513	39.01%	36.36%	41.71%
	>65 years	5	0.38%	0.12%	0.89%	0	0.00%	0.00%	0.00%	5	0.38%	0.12%	0.89%	10	0.76%	0.37%	1.39%
	Empty	0	0.00%	0.00%	0.00%	10	0.76%	0.37%	1.39%	0	0.00%	0.00%	0.00%	10	0.76%	0.37%	1.39%
	Total	1,065	80.99%	78.76%	83.08%	30	2.28%	1.54%	3.24%	220	16.73%	14.75%	18.86%	1,315	100.00%		
Cultural events	<20 years	52	3.95%	2.97%	5.15%	1	0.08%	0.00%	0.42%	4	0.30%	0.08%	0.78%	57	4.33%	3.30%	5.58%
	30–40 years	635	48.29%	45.56%	51.03%	16	1.22%	0.70%	1.97%	74	5.63%	4.44%	7.01%	725	55.13%	52.40%	57.85%
	50–65 years	467	35.51%	32.92%	38.17%	3	0.23%	0.05%	0.67%	43	3.27%	2.38%	4.38%	513	39.01%	36.36%	41.71%
	>65 years	6	0.46%	0.17%	0.99%	0	0.00%	0.00%	0.00%	4	0.30%	0.08%	0.78%	10	0.76%	0.37%	1.39%
	Empty	0	0.00%	0.00%	0.00%	10	0.76%	0.37%	1.39%	0	0.00%	0.00%	0.00%	10	0.76%	0.37%	1.39%
	Total	1,160	88.21%	86.35%	89.91%	30	2.28%	1.54%	3.24%	125	9.51%	7.97%	11.22%	1,315	100.00%		
Airport, port, Train station	<20 years	51	3.88%	2.90%	5.07%	1	0.08%	0.00%	0.42%	5	0.38%	0.12%	0.89%	57	4.33%	3.30%	5.58%
	31–40 years	635	48.29%	45.56%	51.03%	16	1.22%	0.70%	1.97%	74	5.63%	4.44%	7.01%	425	55.13%	52.40%	57.85%
	51–65 years	474	36.05%	33.45%	38.71%	3	0.23%	0.05%	0.67%	36	2.74%	1.92%	3.77%	513	39.01%	36.36%	41.71%
	>65 years	8	0.61%	0.26%	1.19%	0	0.00%	0.00%	0.00%	2	0.15%	0.02%	0.55%	10	0.76%	0.37%	1.39%
	Empty	0	0.00%	0.00%	0.00%	10	0.76%	0.37%	1.39%	0	0.00%	0.00%	0.00%	10	0.76%	0.37%	1.39%
	Total	1,168	88.82%	86.99%	90.47%	30	2.28%	1.54%	3.24%	117	8.90%	7.41%	10.57%	1,315	100.00%		

In contrast, opinions were notably more divided when it came to more sensitive or intimate environments. In educational settings such as schools and universities, support dropped to 45.86%, with a slight majority (51.86%) expressing opposition. A similar pattern emerged for healthcare-related contexts: while 40.38% supported the use of detection dogs in hospitals and 46.77% in nursing homes, a larger proportion of respondents opposed their presence in these settings (57.34% and 50.95%, respectively). These figures may reflect concerns around patient vulnerability, medical privacy, or ethical considerations associated with deploying dogs in spaces involving heightened emotional or physical sensitivity.

Support declined even further for professional and private domains. Only 32.7% supported their use in police or fire departments, while just 12.93% considered workplaces appropriate venues. Hotels received 17.64% approval, and home testing was overwhelmingly rejected, with a mere 3.88% support and 93.84% in opposition. This sharp contrast underscores a reluctance to accept canine testing in contexts perceived as private or intrusive, likely driven by concerns over privacy, appropriateness, and practicality.

### Perceptions of ag-RDTs

Before the concert, 9.65% of participants found Ag-RDTs very reliable, while 50.99% considered it reliable. A neutral stance was taken by 29.05, 9.29% expressed little confidence, and 1.02% reported no confidence in the method.

**Figure 8:**
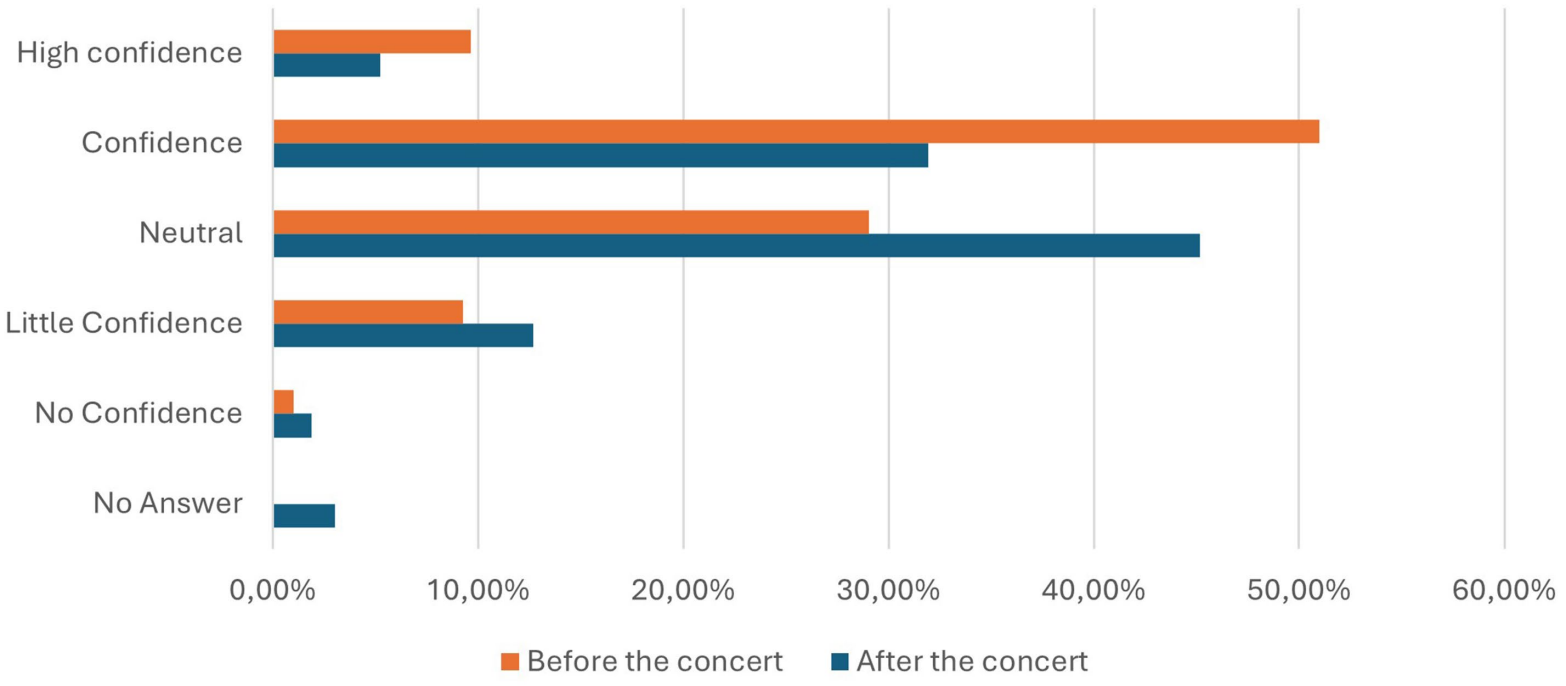
Confidence of the participants in the Ag-RDTs at an official test center before (orange) and after (blue) the concert in percentage.

After the concert, the responses showed slight variations. Only 5.25% rated Ag-RDTs as very reliable, while 31.94% deemed it reliable. Neutral opinions increased to 45.17%, with 12.7% expressing little confidence and 1.9% having no confidence in the testing. Additionally, 3.04% of participants refrained from providing an opinion. The results are presented in Table 6 and visualized in Figure 8.

**Table 6:** Confidence levels in Ag-RDTs in text centers before and after the concerts.

	Pre concert				Post concert			
	Counts	Percentage	Lower CI	Upper CI	Counts	Percentage	Lower Ci	Upper Ci
No answer	0	0.00%	0.00%	0.00%	40	3.04%	2.18%	4.12%
No confidence	42	1.02%	0.73%	1.37%	25	1.90%	1.23%	2.79%
Little confidence	383	9.29%	8.42%	10.21%	167	12.70%	10.95%	14.62%
Neutral	1,198	29.05%	27.67%	30.46%	594	45.17%	42.46%	47.91%
Confidence	2,103	50.99%	49.46%	52.53%	420	31.94%	29.42%	34.54%
High confidence	398	9.65%	8.77%	10.59%	69	5.25%	4.11%	6.59%
Total	4,124	100%			1,315	100%		

### Perceptions of ag-RDT self-testing

Before the concert, 1.41% of participants considered Ag-RDT self-testing very reliable, while 22.28% deemed it reliable. A neutral stance was taken by 39.38%, whereas 28.73% found it somewhat unreliable, and 8.2% expressed no confidence in the method.

**Figure 9:**
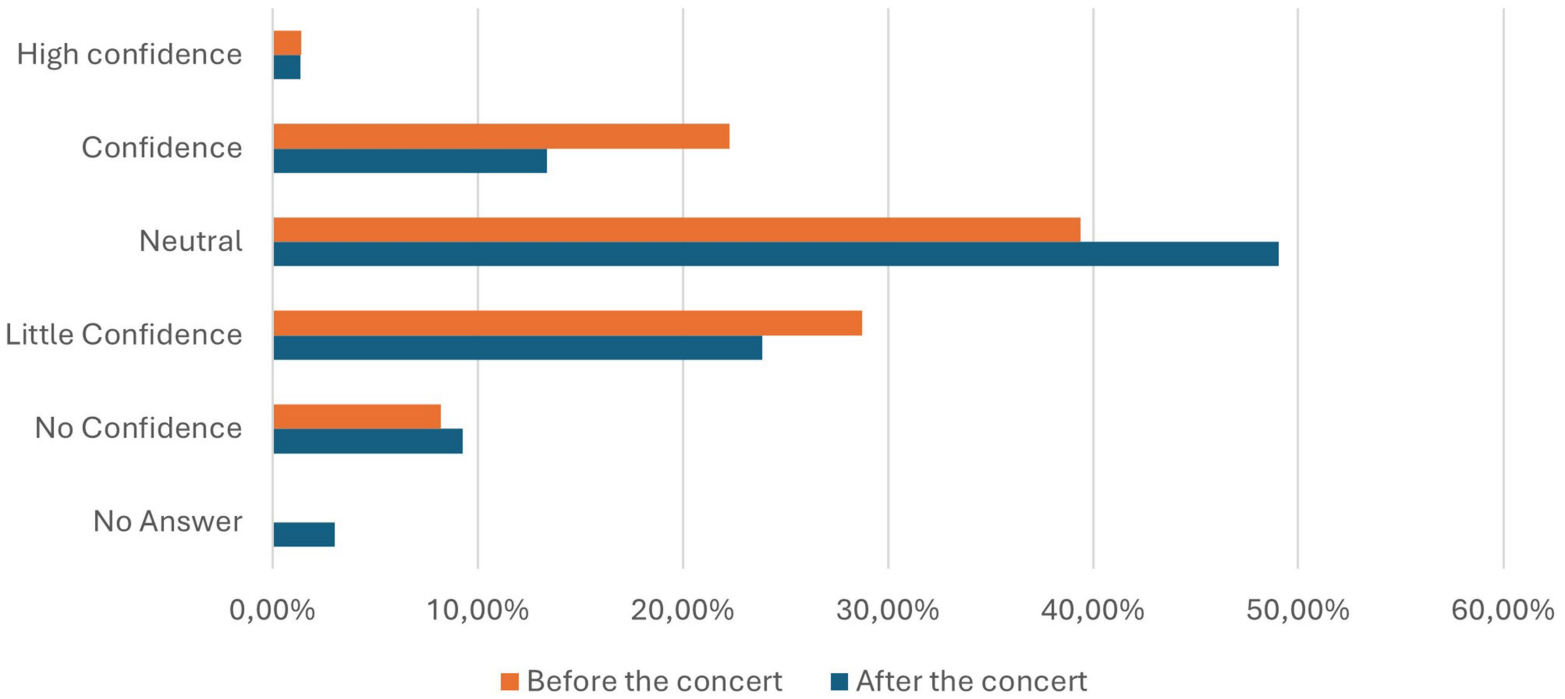
Confidence of the participants in Ag-RD Self-Tests before (orange) and after (blue) the concert in percentage.

After the concert, perceptions showed a slight shift. Only 1.37% of participants considered Ag-RDT self-testing as very reliable, while 13.38% considered it reliable. Neutral opinions increased to 49.05%. In contrast, 23.88% expressed little confidence, and 9.28% reported no confidence. Additionally, 3.04% of participants refrained from answering the question. These results are displayed in Table 7 and illustrated in Figure 9.

**Table 7:** Confidence levels in Ag-RDTs used as self-tests before and after the concerts.

	Pre concert				Post concert			
	Counts	Percentage	Lower CI	Upper CI	Counts	Percentage	Lower CI	Upper CI
No answer	0	0.00%	0.00%	0.00%	40	3.04%	2.18%	4.12%
No confidence	338	8.20%	7.38%	9.08%	122	9.28%	7.76%	10.98%
Little confidence	1,185	28.73%	27.36%	30.14%	314	23.88%	21.60%	26.28%
Neutral	1,624	39.38%	37.88%	40.89%	645	49.05%	46.31%	51.79%
Confidence	919	22.28%	21.02%	23.59%	176	13.38%	11.59%	15.34%
High confidence	58	1.41%	1.07%	1.81%	18	1.37%	0.81%	2.15%
Total	4,124	100%			1,315	100%		

## Discussion

The results of this study provide valuable insights into public perceptions of various COVID-19 testing methods in the context of concert settings, offering essential guidance for shaping public health policies and optimizing event safety planning for a future pandemic. Canine medical detection had a higher acceptance rate than more traditional testing strategies, such as PCR and antigen-based test systems, reflecting a shift toward more innovative, potentially efficient, and less intrusive methods.

The demographics in Table 1 indicate a predominantly young and gender-diverse sample set, which may explain the generally high acceptance of innovative testing approaches like medical detection dogs.

These preferences for different COVID-19 testing methods (Table 2) are visualized in Figures 3A,B and demonstrate a marked increase in the preference for direct sniffing after concert participation (+8.45%), while the preference for sweat samples slightly declined (−6.53%). The sharp drop in preference for Ag-RDTs (−6.64%) suggests decreased trust after experiencing different methods. This shift underscores increased public confidence in canine testing, especially direct sniffing, following real-world exposure.

The statistical analyses presented in Tables 2, 4 confirm a notable increase in both trust and preference for canine detection following the concert. Preference for dog-based testing rose from 70.4% to 72.32%, and trust in detection dogs increased significantly from 82.18% to 94.98% (Table 4), with non-overlapping confidence intervals indicating a statistically meaningful shift. The increase in preference for direct sniffing (from 31.55% to 40%; Table 2) suggests growing acceptance of this less privacy-protective method, likely due to its speed and simplicity. Nevertheless, sweat sample testing remained a widely accepted alternative among participants who preferred indirect contact. These findings suggest that firsthand experience may enhance public confidence in innovative diagnostic approaches such as canine detection.

The study also examined participants’ confidence in PCR testing, which remained consistently high at approximately 87% both before and after the concert (Table 3). Overall, trust in PCR tests showed remarkable stability, with participants maintaining a strong belief in their reliability throughout. This enduring confidence underscores PCR’s continued status as the gold standard in testing, despite the growing interest in canine-based methods.

Confidence in Ag-RDTs at test centers (Table 6) and self-testing (Table 7) plummeted from nearly two out of three participants expressing “confidence” or “high confidence” to just about one out of three after the concert. Similarly, confidence in self-testing dropped from 23.69% to 14.75%. In both cases, “neutrality” increased substantially post-concert, indicating that participants became more hesitant about the reliability of these methods. These findings highlight a decline in trust in antigen-based strategies after experiencing the different testing strategies, further emphasising the growing preference for PCR and innovative methods like canine medical detection (5, 6, 10, 16).

This public shift in trust is consistent with earlier concerns regarding Ag-RDTs’ variable sensitivity and the logistical complexity of PCR workflows. Priyanka, Choudhary et al. (43) emphasized that while PCR is diagnostically superior, its high cost, time requirement, and technical infrastructure limit widespread, real-time application (29). Innovative alternatives such as Point-of-Care Testing and canine detection were thus proposed as scalable, rapid solutions.

### Impact of context and settings

The study further explored the public opinion about the use of detection dogs in different environments (Table 5). Strong support for canine testing was found in high-traffic public settings such as airports, train stations, and ports, where 88.82% of participants considered it an appropriate method. This widespread acceptance indicates that canine testing is viewed as both practical and effective in public settings, particularly at venues with high foot traffic. However, opinions were more divided regarding schools and universities, with support varying by demographic background. Notably, younger participants were more in supportive of the use of detection dogs in educational institutions.

Similarly ambivalent were the responses concerning nursing homes: 46.77% supported the use of detection dogs in these settings, while 50.95% were opposed and 2.28% remained neutral. This split opinion suggests that while some see potential benefits in such environments, concerns about privacy, vulnerability of residents, or appropriateness may temper broader acceptance.

In contrast, the study found limited support for using detection dogs in more personal settings—at home, with only 3.88% of participants considering it appropriate, and in hotels (17.64%). This preference likely stems from privacy concerns and the desire for testing to occur in more controlled, professional environments. The reluctance toward home-based testing likely reflects fears of invasiveness or the discomfort of interacting with dogs in private spaces.

These findings underscore the importance of contextual appropriateness for public acceptance of canine-based screening programs. While detection dogs are generally well accepted in public, anonymous, and security-oriented environments, their deployment in personal, institutional, or domestic settings remains more contentious. Accordingly, the success of future implementations will depend not only on diagnostic performance, but also on alignment with societal expectations, perceived intrusiveness, and logistical feasibility.

### Cultural and historical influences on perceptions

The present study’s results also underscore the profound impact of cultural background on perceptions of canine testing. Notably, our findings concur with those of Grandjean et al. (44), who reported similar trends of support for detection dogs in public settings across multiple countries in 2023. However, cultural attitudes toward canine detection varied significantly, with countries such as Russia and China exhibiting notably lower levels of acceptance (33% and 11.1%, respectively). This disparity can be attributed, in part, to differing societal experiences with animals, particularly in surveillance contexts and regarding dogs as companion animals. Conversely, Western countries such as Germany and France demonstrated higher levels of support (81.1% and 81.4%, respectively), underscoring the complex interplay between cultural background and perceptions of canine testing.

In Germany, the use of detection dogs may face specific challenges due to historical associations with animals in surveillance and control. During World War II and under the East German regime, dogs, particularly German Shepherds, were used by authorities for state control and surveillance (45–47). This could evoke negative memories, particularly among older generations. Notwithstanding these historical associations, 40% of participants in the study expressed a preference for direct sniffing, while the sweat sample variant received an acceptance rate of 32.2%. The sweat sample method, although slightly less time-efficient than direct sniffing, is less invasive and more considerate of privacy and individual preferences, such as concerns about allergies, fear of dogs, or discomfort with direct contact and data protection. Moreover, the effort involved in collecting a sweat sample is comparable to that of an Ag-RDT, avoiding the more uncomfortable aspects associated with sampling from the nose or throat, making it a more acceptable alternative for many.

Potential sampling bias may exist, as participants were informed in advance about the testing modalities used, which may have increased their openness to innovative testing methods. Furthermore, as concert-goers, the participants might inherently be more receptive to such approaches. Additionally, individuals attending an event featuring canine testing are likely more predisposed to accept this method compared to the general population. While the findings provide valuable insights into public attitudes toward canine testing, they should be interpreted with caution and not generalized to the broader German population without further studies involving a more representative sample.

### Impact on public health policy

The study’s findings are crucial for shaping public health policy, particularly when developing testing strategies for large public events. The high level of support for canine testing suggests it could offer a viable alternative or complement to traditional methods like PCR and Ag-RDTs, especially in situations where speed, cost, and efficiency are key. The increased trust in detection dogs—especially after direct exposure at public events—demonstrates their potential for mass testing at large gatherings such as concerts, festivals, and other crowded occasions.

In comparison to PCR tests, which are more time-consuming and expensive (40, 48), detection dogs offer a faster and more efficient solution for high-traffic events, providing real-time detection that accelerates the testing process (11, 36). When scaled to large test numbers, detection dogs also prove to be more cost-effective than Ag-RDTs, and significantly cheaper than PCR testing (40).

Additionally, their sensitivity is often higher than that of laboratory tests, making them a reliable alternative for mass screenings, where quick and accurate results are essential (6, 38, 39).

However, as Priyanka, Choudhary et al. (43) argue, testing methods alone are insufficient without effective rollout and implementation strategies (41). Even highly efficacious interventions, such as vaccines, must be deployed rapidly and broadly to achieve the desireds population-level impact. This insight similarly implies to canine testing: beyond demonstrating effectiveness, success depends on public trust, strategic communication, and logistical feasibility.

These findings imply that a combination of testing methods may prove optimal in various contexts.

Explosive Detection Dog teams are already used at mass events, and the DIN SPEC 77201 was developed to provide a recognized quality standard and improved training methods (49).

Establishing EU guidelines for medical detection dogs, particularly for pandemic-related mass screenings, would ensure their reliable and consistent deployment (33), much like the standards for EDDs. Standardized regulations are crucial for ensuring the acceptance and effectiveness of medical detection dogs across various settings, maintaining public confidence, and supporting the broader adoption of canine-based testing.

## Conclusion

This study provides a comprehensive assessment of public attitudes in Germany toward various SARS-CoV-2 testing methods, with a particular focus on the implementation of medical detection dogs in a real-world setting. The primary contribution lies in demonstrating the high acceptance and trust in canine detection among concert attendees, particularly following direct exposure to the testing procedure. Participants’ confidence in detection dogs remained consistently high throughout the study and ultimately surpassed that of antigen-based tests, whose perceived reliability declined after the events. While PCR continued to be regarded as reliable, detection dogs were increasingly seen as a comparable and practical alternative.

Additional results indicated that canine-based screening was not only favorably received but also considered suitable for deployment in high-traffic public spaces, such as airports and train stations. Its non-invasive nature, rapid turnaround time, and relatively low cost represent significant advantages over conventional diagnostic methods. However, the study also identified limitations, including more cautious attitudes toward dog-based testing in sensitive environments such as nursing homes or private settings, as well as logistical challenges related to scaling up animal-based diagnostics.

By combining structured pre- and post-event data collection with practical testing under controlled event conditions, this study illustrates the operational feasibility and societal acceptance of detection dogs in mass screening scenarios. These findings provide valuable evidence supporting the integrating non-traditional testing methods into future pandemic preparedness plans. For the broader community, the results advocate for the development of flexible, trusted, and cost-effective testing strategies that enhance public health responsiveness while maintaining social and cultural activities. The standardization of protocols and regulatory frameworks for medical detection dogs could further increase their utility as a reliable tool in public health surveillance.

## Data Availability

The datasets presented in this article are not readily available because the original dataset contains private and personally identifiable information about the study participants, including names, contact details, and medical history. In accordance with data protection regulations and ethical guidelines, these data cannot be shared publicly. Requests to access the datasets should be directed to friederike.twele@tiho-hannover.de.

## References

[1] Han E Tan MMJ Turk E Sridhar D Leung GM Shibuya K. Lessons learnt from easing COVID-19 restrictions: an analysis of countries and regions in Asia Pacific and Europe. Lancet. (2020) 396:1525–34. doi: 10.1016/S0140-6736(20)32007-9, PMID: 32979936 PMC7515628

[2] Dzurova D Kveton V. How health capabilities and government restrictions affect the COVID-19 pandemic: cross-country differences in Europe. Appl Geogr. (2021) 135:102551. doi: 10.1016/j.apgeog.2021.102551, PMID: 34456395 PMC8382583

[3] Zhang R Ji H Pang Y Suo L. The impact of COVID-19 on cultural industries: an empirical research based on stock market returns. Front Public Health. (2022) 10:806045. doi: 10.3389/fpubh.2022.806045, PMID: 36187644 PMC9523150

[4] Jeannotte MS. When the gigs are gone: valuing arts, culture and media in the COVID-19 pandemic. Soc Sci Humanit Open. (2021) 3:1–7. doi: 10.1016/j.ssaho.2020.100097

[5] Jegerlehner S Suter-Riniker F Jent P Bittel P Nagler M. Diagnostic accuracy of a SARS-CoV-2 rapid antigen test in real-life clinical settings. Int J Infect Dis. (2021) 109:118–22. doi: 10.1016/j.ijid.2021.07.010, PMID: 34242764 PMC8260496

[6] Khandker SS Nik Hashim NHH Deris ZZ Shueb RH Islam MA. Diagnostic accuracy of rapid antigen test kits for detecting SARS-CoV-2: a systematic review and meta-analysis of 17,171 suspected COVID-19 patients. J Clin Med. (2021) 10:3493. doi: 10.3390/jcm10163493, PMID: 34441789 PMC8397079

[7] Kubina R Dziedzic A. Molecular and serological tests for COVID-19 a comparative review of SARS-CoV-2 coronavirus laboratory and point-of-care diagnostics. Diagnostics. (2020) 10:434. doi: 10.3390/diagnostics10060434, PMID: 32604919 PMC7345211

[8] Liu R Fu A Deng Z Li Y Liu T. Promising methods for detection of novel coronavirus SARS-CoV-2. View. (2020) 1:e4. doi: 10.1002/viw2.4, PMID: 38607796 PMC7169335

[9] Chaber AL Hazel S Matthews B Withers A Alvergnat G Grandjean D. Evaluation of canine detection of COVID-19 infected individuals under controlled settings. Transbound Emerg Dis. (2022) 69:e1951–8. doi: 10.1111/tbed.14529, PMID: 35316576 PMC9115492

[10] Jendrny P Schulz C Twele F Meller S von Köckritz-Blickwede M Osterhaus A. Scent dog identification of samples from COVID-19 patients - a pilot study. BMC Infect Dis. (2020) 20:536. doi: 10.1186/s12879-020-05281-3 32703188 PMC7376324

[11] Kantele A Paajanen J Turunen S Pakkanen SH Patjas A Itkonen L. Scent dogs in detection of COVID-19: triple-blinded randomised trial and operational real-life screening in airport setting. BMJ Glob Health. (2022) 7:e008024. doi: 10.1136/bmjgh-2021-008024, PMID: 35577391 PMC9108438

[12] Bevan I Stage Baxter M Stagg HR Street A. Knowledge, attitudes, and behavior related to COVID-19 testing: a rapid scoping review. Diagnostics. (2021) 11:1685. doi: 10.3390/diagnostics11091685, PMID: 34574026 PMC8472251

[13] Street A Lee SJ Bevan I. The hidden burden of medical testing: public views and experiences of COVID-19 testing as a social and ethical process. BMC Public Health. (2022) 22:1837. doi: 10.1186/s12889-022-14217-2, PMID: 36180839 PMC9524338

[14] Bleckwenn M. Screening auf COVID-19 mittels Fiebermessung? MMW Fortschr Med. (2020) 162:21–2. doi: 10.1007/s15006-020-4614-2 PMC7735181 33319290

[15] Datenschutzokonferenz D. Einsatz von Wärmebildkameras bzw. elektronischer Temperaturerfas- sung im Rahmen der Corona-Pandemie. Datenschutzkonferenz.

[16] Lee J Song JU Shim SR. Comparing the diagnostic accuracy of rapid antigen detection tests to real time polymerase chain reaction in the diagnosis of SARS-CoV-2 infection: a systematic review and meta-analysis. J Clin Virol. (2021) 144:104985. doi: 10.1016/j.jcv.2021.104985, PMID: 34560340 PMC8444381

[17] Weiss C. PCR tests and rapid antigen tests-how reliable are they? Notf Rett Med. (2022) 25:48–50. doi: 10.1007/s10049-021-00977-8, PMID: 35043046 PMC8756741

[18] Floriano I Silvinato A Bernardo WM Reis JC Soledade G. Accuracy of the polymerase chain reaction (PCR) test in the diagnosis of acute respiratory syndrome due to coronavirus: a systematic review and meta-analysis. Rev Assoc Med Bras. (1992) 66:880–8. doi: 10.1590/1806-9282.66.7.880, PMID: 32844930

[19] infektionsschutz.de. PCR-Test: Goldstandard unter den Corona-Tests (2023).

[20] Xu M Wang D Wang H Zhang X Liang T Dai J. COVID-19 diagnostic testing: technology perspective. Clin Transl Med. (2020) 10:e158. doi: 10.1002/ctm2.158, PMID: 32898340 PMC7443140

[21] Madadelahi M Agarwal R Martinez-Chapa SO Madou MJ. A roadmap to high-speed polymerase chain reaction (PCR): COVID-19 as a technology accelerator. Biosens Bioelectron. (2024) 246:115830. doi: 10.1016/j.bios.2023.115830, PMID: 38039729

[22] Gopaul R Davis J Gangai L Goetz L. Practical diagnostic accuracy of nasopharyngeal swab testing for novel coronavirus disease 2019 (COVID-19). West J Emerg Med. (2020) 21:1–4. doi: 10.5811/westjem.2020.8.48420, PMID: 33052811 PMC7673872

[23] Dogan U Karatas G Mihrap Ilter S. Factors affecting procedural pain and discomfort experienced by individuals during nasopharyngeal swabbing: a cross-sectional study. Florence Nightingale J Nurs. (2024) 32:215–20. doi: 10.5152/FNJN.2024.22289 39530582 PMC11562252

[24] Marra P Colacurcio V Bisogno A De Luca P Calvanese M Petrosino M. Evaluation of discomfort in nasopharyngeal swab specimen collection for SARS-CoV-2 diagnosis. Clin Ter. (2021) 172:448–52. doi: 10.7417/CT.2021.2357, PMID: 34625778

[25] Whelehan P Evans A Wells M Macgillivray S. The effect of mammography pain on repeat participation in breast cancer screening: a systematic review. Breast. (2013) 22:389–94. doi: 10.1016/j.breast.2013.03.003, PMID: 23541681

[26] Tanja A Treschan TS Kober A Fleischmann E Birkenberg B Petschnigg B. The influence of protocol pain and risk on patients’ willingness to consent for clinical studies: a randomized trial. Anesth Analg. (2003) 96:498–506. doi: 10.1097/00000539-200302000-00037 12538203

[27] Federal Ministry of Health. Coronavirus-Testverordnung – TestV, (2021).

[28] Grosch J Schomakers L Grohmann M Witte AK. COVID-19-Bürgertests im Sommer 2021: Demografie und Motivation der Testpersonen. Epidemiol Bull, (2022) 15:3–13. doi: 10.25646/9798

[29] Priyanka Choudhary OP Singh I. Diagnosis of SARS-CoV-2: a review on the current scenario and future outlook. Acta Virol. (2020) 64:396–408. doi: 10.4149/av_2020_40 32985200

[30] Dickey T Junqueira H. COVID-19 scent dog research highlights and synthesis during the pandemic of December 2019-April 2023. J Osteopath Med. (2023) 123:509–21. doi: 10.1515/jom-2023-0104, PMID: 37452676

[31] Jendrny P Twele F Meller S Osterhaus A Schalke E Volk HA. Canine olfactory detection and its relevance to medical detection. BMC Infect Dis. (2021) 21:838. doi: 10.1186/s12879-021-06523-8, PMID: 34412582 PMC8375464

[32] Lippi G Mattiuzzi C Henry BM. Are sniffer dogs a reliable approach for diagnosing SARS-CoV-2 infection? Diagnosis. (2021) 8:446–9. doi: 10.1515/dx-2021-0034, PMID: 33873262

[33] Meller S Al Khatri MSA Alhammadi HK Alvarez G Alvergnat G Alves LC. Expert considerations and consensus for using dogs to detect human SARS-CoV-2-infections. Front Med. (2022) 9:1015620. doi: 10.3389/fmed.2022.1015620, PMID: 36569156 PMC9773891

[34] Pirrone F Piotti P Galli M Gasparri R La Spina A Spaggiari L. Sniffer dogs performance is stable over time in detecting COVID-19 positive samples and agrees with the rapid antigen test in the field. Sci Rep. (2023) 13:3679. doi: 10.1038/s41598-023-30897-1, PMID: 36872400 PMC9985821

[35] Ten Hagen NA Twele F Meller S Jendrny P Schulz C von Köckritz-Blickwede M. Discrimination of SARS-CoV-2 infections from other viral respiratory infections by scent detection dogs. Front Med. (2021) 8:749588. doi: 10.3389/fmed.2021.749588, PMID: 34869443 PMC8636992

[36] Ten Hagen NA Twele F Meller S Wijnen L Schulz C Schoneberg C. Canine real-time detection of SARS-CoV-2 infections in the context of a mass screening event. BMJ glob. Health. (2022) 7:e010276. doi: 10.1136/bmjgh-2022-010276, PMID: 36368765 PMC9659709

[37] WHO Consultation on the use of trained dogs for screening COVID-19 cases WHO (2020)

[38] Hag-Ali M AlShamsi AS Boeijen L Mahmmod Y Manzoor R Rutten H. The detection dogs test is more sensitive than real-time PCR in screening for SARS-CoV-2. Commun Biol. (2021) 4:686. doi: 10.1038/s42003-021-02232-9, PMID: 34083749 PMC8175360

[39] Grandjean D Elie C Gallet C Julien C Roger V Desquilbet L. Diagnostic accuracy of non-invasive detection of SARS-CoV-2 infection by canine olfaction. PLoS One. (2022) 17:e0268382. doi: 10.1371/journal.pone.0268382, PMID: 35648737 PMC9159600

[40] Mutesa L Misbah G Remera E Ebbers H Schalke E Tuyisenge P. Use of trained scent dogs for detection of COVID-19 and evidence of cost-saving. Front Med. (2022) 9:1006315. doi: 10.3389/fmed.2022.1006315, PMID: 36530913 PMC9751420

[41] Priyanka COP Singh I. Making sound public health policy decisions for COVID-19 vaccination: vaccine effectiveness, safety, affordability, programmatic logistics and roll-out globally. J Travel Med. (2021) 5:taab031. doi: 10.1093/jtm/taab031 PMC7989503 33690836

[42] Priyanka COP. Vaccine efficacy against COVID-19: a foresight on the host-associated factors. J Formos Med Assoc. (2021) 120:1405–7. doi: 10.1016/j.jfma.2020.11.021 33380377 PMC7832210

[43] Choudhary OP Priyanka Ahmed JQ Mohammed TA Singh I Rodriguez-Morales AJ. Heterologous prime-boost vaccination against COVID-19: is it safe and reliable? Hum Vaccin Immunother. (2021) 17:5135–8. doi: 10.1080/21645515.2021.2007015 34898381 PMC8726007

[44] Grandjean D Hache F Gallet C Bacqué H Blondot M Desquilbet L. Acceptation par le public de la détection olfactive canine de la COVID-19: à propos d’une enquête internationale avant redéploiement. Bull Acad Vet Fr. (2023) 176:336–49. doi: 10.3406/bavf.2023.18303

[45] Besser-Seuß A.. Schäferhund als Waffe in der DDR und Rassenikone zur NS-Zeit. MDR.DE: MDR.DE; 2024 27.11.2024.

[46] Perz B. müssen zu reißenden Bestien erzogen werden: Der Einsatz von Hunden zur Bewachung in den Konzentrationslagern. Dachauer Hefte. (1996) 12:20.

[47] the_wall_museum. Tiere im Grenzstreifen - Wall Museum. (2018).

[48] Gupta N Augustine S Narayan T O'Riordan A Das A Kumar D. Point-of-care PCR assays for COVID-19 detection. Biosensors. (2021) 11:141. doi: 10.3390/bios11050141, PMID: 34062874 PMC8147281

[49] Becher C Kaul P-M. Enhancing quality in commercially used explosives detection dogs. Int J Saf Secur Eng. (2024) 14:321–7. doi: 10.18280/ijsse.140201

