# Supplementary material for: Public perception of medical detection dogs and other COVID-19 testing strategies
Source: Front Public Health. 2025 Sep 4;13:1641243. doi: 10.3389/fpubh.2025.1641243 (PMC12443801; doi:10.3389/fpubh.2025.1641243)
Supplement: Supplementary file 1 [file Data_Sheet_1.PDF]

Public Perception of Medical Detection Dogs and other COVID-19 Testing Strategies

Karolina Zacharias[1], Sebastian Meller[1,2], Nele ten Hagen[1], Holger A. Volk[1,2], Friederike Twele[1,2*]

[1]Department of Small Animal Medicine and Surgery, University of Veterinary Medicine Hannover, Hannover, Germany
[2]Center for Systems Neuroscience Hannover, Hannover, Germany
*** Correspondence:**
Friederike Twele
Friederike.twele@tiho-hannover.de
Department of Small Animal Medicine and Surgery
University of Veterinary Medicine Hannover
Bünteweg 9
30559 Hannover
30559 Hannover

1. Preliminary Questionnaire

Personal Information

- Last Name, First Name

- Address

- Email

- Phone Number

- Date of Birth

- Gender (male/female/diverse)

SARS-CoV-2 Vaccination Status:

- No vaccination

- Incomplete vaccination

- Full vaccination

Vaccine received (1st dose):

- Comirnaty (BioNTech)

- COVID-19 Vaccine Moderna
- Vaxzevria (AstraZeneca)
- COVID-19 Vaccine Janssen (Johnson & Johnson)
- Not applicable

Vaccine received (2nd dose):

- Comirnaty (BioNTech)
- COVID-19 Vaccine Moderna
- Vaxzevria (AstraZeneca)
- Not applicable

Have you been infected with SARS-CoV-2?

- Yes
- No
- I don’t know / no confirmed diagnosis

If “Yes”:

- Date of acute illness
- Symptoms: (free text)
- Have you been diagnosed with Long COVID?
  - Yes
  - No

  If “Yes”:

    - Symptoms: (free text)
    - Date of Long COVID diagnosis

Do you suffer from any chronic illnesses (e.g., diabetes, high blood pressure)?

- Yes

- No

If “Yes”:

- Free text: ___________

Perception Questions (Scale: 1 = Fully agree / absolute confidence, 5 = Fully disagree / no confidence)

1. Do you believe that dogs should be used for COVID-19 testing?

2. How much confidence do you have in the results of a COVID-19 sniffer dog test?

3. How much confidence do you have in the results of a COVID-19 PCR test?

4. How much confidence do you have in the results of a COVID-19 antigen test (official testing center)?

5. How confident are you in the results of a COVID-19 self-test?

6. Which COVID-19 test would you prefer upon arrival at an airport or port?

  - Sweat sample test by a sniffer dog

  - PCR test via nasal swab

  - Direct sniffing by a sniffer dog

  - Another rapid test

2. Follow-Up Questionnaire

1. You are:

  ☐ Male ☐ Female ☐ Diverse

2. Age group:

  ☐ Under 20 years ☐ 21–40 years ☐ 41–65 years ☐ Over 65 years

3. How satisfied were you with the sweat sample test procedure using sniffer dogs at the event venue?

  (Scale: 1 = Not at all satisfied, 5 = Absolutely satisfied)

4. Do you believe dogs should be used to test sweat samples for SARS-CoV-2 detection?

  (Scale: 1 = Strongly disagree, 5 = Strongly agree)

5. In which settings would you like to see sniffer dogs used for COVID-19 detection? (Multiple answers possible)

☐ None

☐ Airport, train station, seaport

☐ Schools, universities

☐ Medical personnel, fire department, police, armed forces

☐ Nursing homes

☐ Sporting events

☐ Cultural events

☐ Conferences

☐ Workplace

☐ Hotels

☐ At home

6. Would you be willing to be sniffed by a dog for COVID-19 detection?

☐ Yes ☐ No

7. If “Yes”:

☐ But only without physical contact

☐ I don’t mind physical contact

8. How much confidence do you have in the following COVID-19 test methods?

(Scale: 1 = No confidence, 5 = Full confidence)

Test Type	1	2	3	4	5
Sniffer dog test	☐	☐	☐	☐	☐
PCR test	☐	☐	☐	☐	☐
Antigen test (test center)	☐	☐	☐	☐	☐

| Self-test | ☐ | ☐ | ☐ | ☐ | ☐ |

9. Which COVID-19 test would you prefer?

☐ Sweat sample test by a sniffer dog

☐ PCR test via nasal swab

☐ Rapid antigen test via nasal swab

☐ Direct sniffing by a sniffer dog
